# Inter- and intra-observer agreement in ultrasound diagnosis of steatotic liver disease: implications for screening in resource-limited settings

**DOI:** 10.1038/s41598-025-07862-1

**Published:** 2025-08-14

**Authors:** Maria Spencer-Sandino, Maya Balakrishnan, David Wynne, Ilona Argirion, Paz Cook, Vanessa Van De Wyngard, Noldy Mardones, Ruth Pfeiffer, Allan Hildesheim, Catterina Ferreccio, Jill Koshiol

**Affiliations:** 1 Escuela de Salud Pública, Facultad de Medicina, Pontificia Universidad Católica de Chile, 8330077 Santiago, RM Chile; 2 Section of Gastroenterology and Hepatology, Department of Internal Medicine, Baylor College of Medicine, Houston, TX 77030 USA; 3 Center for Innovations in Quality, Effectiveness, and Safety (IQuESt), Michael E. DeBakey Veterans Affairs Medical Center, Houston, TX 77021 USA; 4 Department of Radiology, Ben Taub Hospital, Houston, TX 77030 USA; 5 Human Science Academic Department, Georgetown University, Washington, DC 20057 USA; 6 Gillings School of Global Public Health, University of North Carolina at Chapel Hill, Chapel Hill, NC 27599 USA; 7 Hospital Dr. Hernán Henríquez Aravena, 4781151 Temuco, Chile; 8 Division of Cancer Epidemiology and Genetics, Infections and Immunoepidemiology Branch, NIH/NCI, 9609 Medical Center Dr, Rockville, MD 20850 USA; 9 Instituto de Salud Publica de Chile, ISP, Santiago, Chile

**Keywords:** Steatotic liver disease, Ultrasound, Inter-observer agreement, Diagnostics, Non-alcoholic fatty liver disease, Epidemiology

## Abstract

Steatotic liver disease (SLD), which is associated with increased risk of cancer-related mortality, needs timely and cost-effective detection. Although liver biopsy remains the diagnostic gold standard, its invasiveness and high-cost limit widespread use. Ultrasound is a practical and affordable alternative. We evaluated inter- and intra-observer agreement for ultrasound-based diagnosis of SLD using images from the Chile Biliary Longitudinal Study (Chile BiLS), a cohort of women with gallstones. These women have a high burden of obesity and related metabolic disorders, putting them at higher risk for SLD. A radiologist (observer 1) reviewed a randomly selected subset of 425 baseline images and compared them with the original readings from Chile BiLS radiology technicians. To assess intra-observer reproducibility, observer 1 reanalyzed 34 blinded duplicates, and two Chile BiLS radiology technicians (observers 2 and 3) independently reviewed these images. Observer 2 then re-reviewed the 34 images to assess intra-observer agreement. Agreement was analyzed using kappa and percent agreement. Observer 1 had slight inter-observer agreement (kappa: 0.12; 95% CI 0.08–0.15, *p* < 0.001; percent agreement: 41.0%), while observers 2 and 3 showed fair agreement (kappa: 0.29: 95% CI 0.11–0.58, *p* < 0.05; percent agreement: 64.7% and kappa: 0.32: 95% CI 0.06–0.58, *p* < 0.05; percent agreement: 63.6%, respectively). Intra-observer agreement was moderate for observer 1 (kappa: 0.45; 95% CI 0.08–0.82, *p* < 0.05; percent agreement: 81.3%), and substantial for observer 2 (kappa: 0.64; 95% CI 0.37–0.90, *p* < 0.001; percent agreement: 81.8%). Our findings highlight variability in ultrasound interpretation, underscoring the necessity of inter- and intra-observer comparisons for optimal diagnosis and quality control to enhance diagnostic consistency in high-risk populations.

## Introduction

Steatotic liver disease (SLD) is a chronic condition associated with an increased risk for cancer-related mortality, mainly for hepatocellular carcinoma^1–3^. SLD is defined as abnormal triglyceride accumulation within the hepatocyte, known as hepatic steatosis^2^. The term SLD, implemented in June 2023, encompasses a variety of disease subcategories, including metabolic-associated steatotic liver disease (MASLD), metabolic and alcohol-associated liver disease (MetALD), alcohol-associated liver disease (ALD), and several rarer subtypes with specific or cryptogenic etiologies^3^. Previously known as non-alcoholic fatty liver disease (NAFLD), MASLD is the most prevalent form of SLD^4^.

Over 25%-30% of the global population has MASLD^5,6^. Chronic conditions such as obesity, type 2 diabetes, hypertension, and cardiometabolic conditions are associated with MASLD^7^. Given the increasing rates of obesity and type 2 diabetes in Latin America, the prevalence of MASLD is believed to be underestimated and underreported^8^. High rates of MASLD have been reported in Chile, with prevalence estimates ranging from 23% in 2000 among those over 18 years old ^9^ to 47.5% in 2019 among those 38–74 years old^10^.

The gold standard for diagnosing hepatic steatosis is liver biopsy^11^. However, its invasive nature and high cost make it less viable for primary healthcare^12^. Current guidelines in the United States, Europe, and Latin America recommend using alternative imaging technology, such as elastography, to diagnose liver steatosis^6,13,14^. Unfortunately, this technology is not routinely available worldwide due to its high cost^14^. Ultrasound is the most commonly recommended alternative to elastography due to its practicality, low cost, safety, and wide availability^13–15^. Even though ultrasound is a feasible alternative, this technique has limitations. Ultrasound is highly operator-dependent, explaining the variability in sensitivity (between 60 and 94%) and specificity (between 84 and 95%), which are also influenced by the degree of steatosis in the liver^12^. Additionally, the detection of steatosis by ultrasound is affected by visceral fat, hindering the accuracy of this technology among obese patients^12,14^. Training and quality controls are necessary to achieve a precise technique, as the interpretation of the image can affect the diagnosis of steatosis.

Chile has a high prevalence of MASLD^10^ and high rates of gallstone disease^16^, which has been associated with MASLD^17,18^. A previous report in a Chilean population of adults aged 38 to 74 found the prevalence of gallstones to be twice as high in women compared to men (40% vs 20%)^10^. Additionally, studies have observed that women with MASLD over the age of 50 have a higher risk of developing advanced fibrosis than men^19^. The Chile Biliary Longitudinal Study (Chile BiLS), a prospective cohort of women with gallstones, collected ultrasound images that can be used to assess the prevalence of hepatic steatosis in this population^18^. Chile BiLS used radiology technicians, rather than radiologists, for ultrasound interpretation, following the recommendations of most governments and the World Health Organization to involve highly qualified health workers, such as radiology technicians, in clinical diagnosis, given the lack of specialized physicians in low- and middle-income countries^20^. Despite the implementation of this approach in diverse countries, concerns about the precision of the exams remain^20^. Consequently, we reviewed a subset of baseline ultrasound images to ascertain the presence/absence of SLD and to evaluate interobserver and intraobserver agreement in image assessments between the radiology technicians from the Chile BiLS cohort and a university-based radiologist with experience in liver images in the United States.

## Methods

### Study population and design

This cross-sectional analysis used data from Chile BiLS, an ongoing prospective cohort initiated in 2016^18^. The cohort includes 4338 women aged 50–74 residing in the Cautín Province, Araucanía region. Participants underwent baseline, year-2, and year-4 visits, during which demographic, history of type 2 diabetes or use of medication, blood pressure, history of hypertension or use of medication, physical examination (height, weight, and waist circumference), blood samples, and ultrasound data were collected^18^. Chile BiLS radiology technicians conducted ultrasound examinations; these technicians had a specialty in radiology and underwent formal ultrasonography training conducted by a radiologist who worked in the cohort. This training was based on the standardized radiological guidelines for ultrasound interpretation^21,22^. All ultrasound reports were reviewed and signed by a radiologist.

**Fig. 1:**
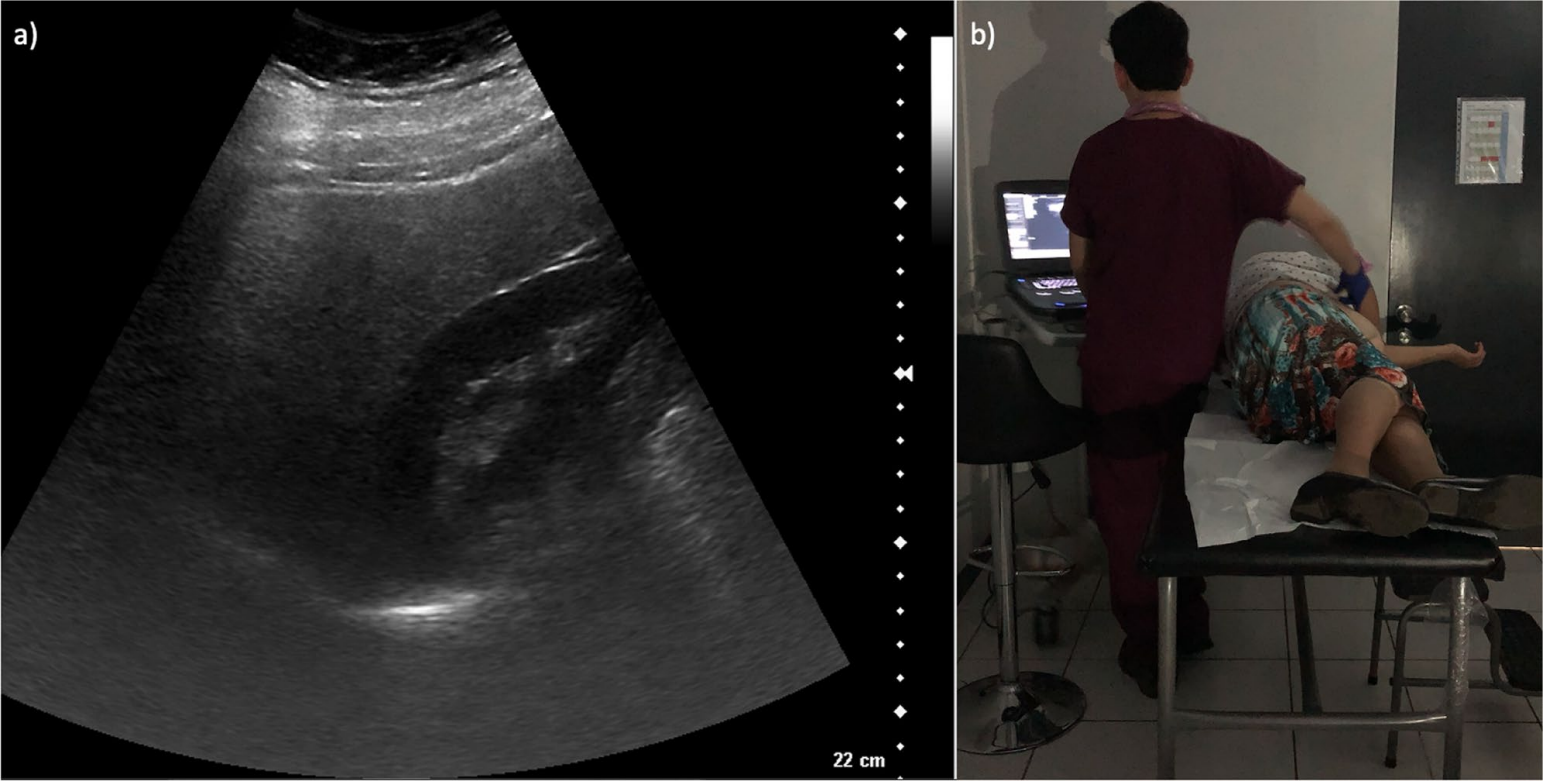
(**a**) Still ultrasound image (static photograph) of the liver demonstraing that the liver and the kidney were captured on the same plane. (**b**) A Chile BiLS technician performing an ultrasound on a participant.

**Fig. 2:**
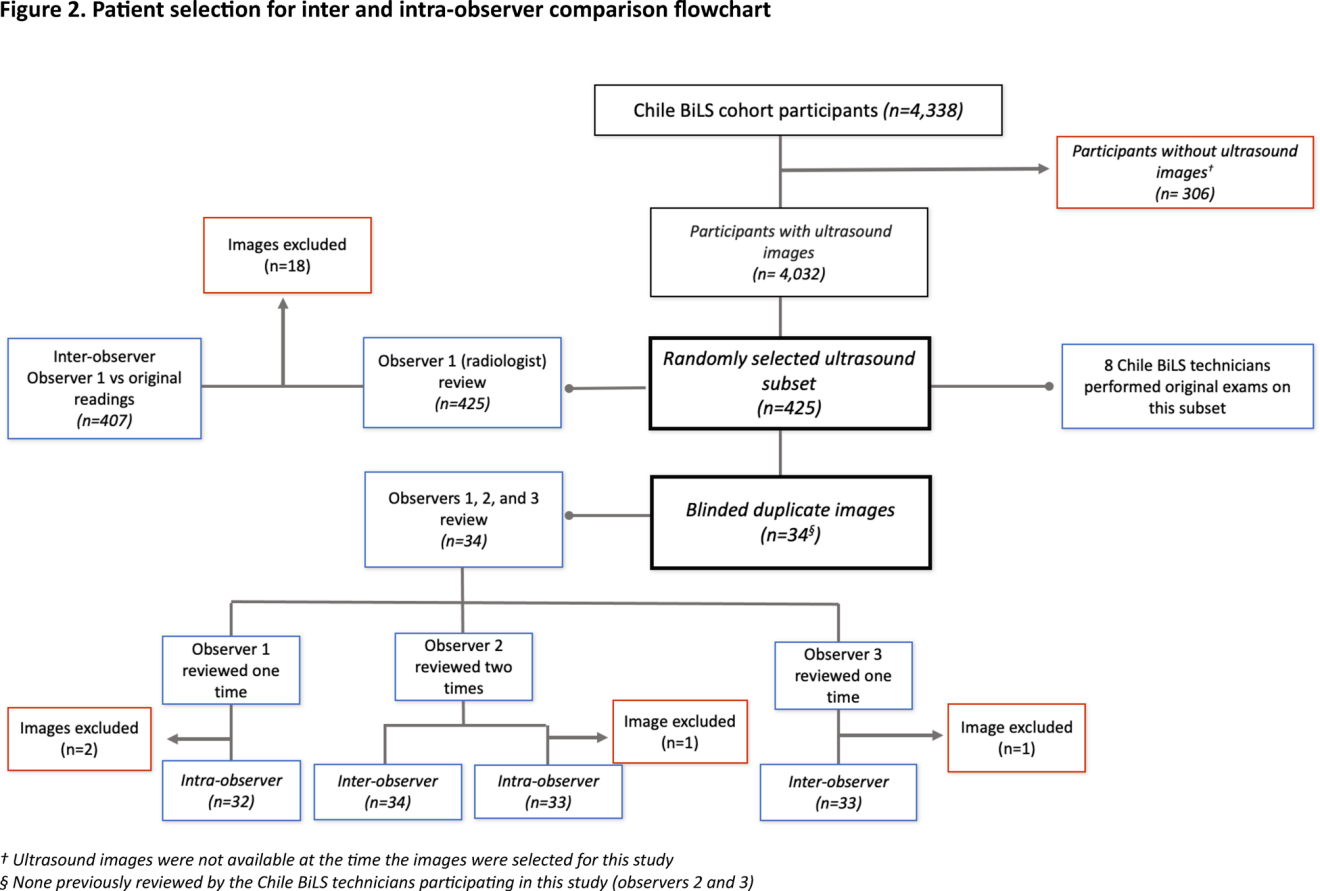
Patient selection for inter and intra-observer comparison flowchart.

Using computer-generated randomization, we selected a random subset of 425 (~ 10%) participants from the 4032 participants with baseline images available in 2019 (Fig. 1) to assess agreement in identifying liver steatosis. The evaluation involved comparing the findings for hepatic steatosis from the original ultrasound examinations done by eight Chile BiLS radiology technicians (original readings) with the interpretation of a radiologist from Baylor College of Medicine (observer 1), who reviewed still digital ultrasound images taken during the examination. Blinded duplicate images from 34 randomly selected participants (10% of the original subset, which was slightly lower than the 425 finally included) were included to assess reliability. This duplicated set of images was re-evaluated by observer 1 and by two Chile BiLS radiology technicians (observers 2 and 3). Observers 2 and 3 were part of the eight radiology technicians who generated the original readings and contributed four of the readings (three from observer 2 and one from observer 3) to the random set of 425. However, they did not conduct any of the examinations for the participants included in the set of 34 with blinded duplicate images. Finally, observer 2 re-evaluated the duplicated set of 34 images to assess intraobserver agreement (Fig. 2).

### Diagnosis of liver steatosis

Liver steatosis was ascertained through hepatobiliary ultrasonography using Siemens ACUSON P500™ and P300™ portable ultrasound machines. During the examination, when the original images were taken, the radiology technician documented and recorded both biliary and liver findings. The radiology technicians were available to explore different angles to determine biliary and liver findings, obtaining a set of still images for each patient. When a radiology technician had concerns (e.g., ultrasound could not be performed due to abdominal adiposity or other reasons, or radiology technicians were not certain of the observed features), findings were discussed with a Chilean radiologist. In the current analysis, the radiology technicians and radiologist identified liver steatosis by comparing the echogenicity of the liver related to the kidney. Observers reviewed the liver parenchyma, and when it was more echogenic than the renal cortex (“brighter”), then steatosis was confirmed^23^. To determine the degree of steatosis, observers compared whether the right hemidiaphragm could be resolved separately from the liver dome. If the structures were resolvable, they were classified as “mild,” and if they could not be distinguished, they were classified as “moderate/severe”^23^. None of the images had abnormally echogenic kidneys, and participants had no history of renal disease. In the original readings, hepatic steatosis was categorized into one of four categories: absent, mild, moderate, or severe. Upon re-review, the radiologist and both Chilean radiology technicians categorized the degree of hepatic steatosis into three categories: none, mild, moderate/severe.

### Statistical analysis

Intra- and interobserver agreement were evaluated according to the presence versus absence of steatosis (categorized as yes/no) and the severity of steatosis (none, mild, moderate/severe). Interobserver agreement for observer 1 (radiologist) was performed by comparing the review of the 425 randomly selected images (407 in the final review, as described below and in Fig. 2) versus the original readings. For observers 2 and 3 (radiology technicians), review of the duplicate set of 34 participants against the original readings. Concordance between the original readings and observers 1, 2, and 3 was quantified by both percent agreement and kappa values, using Cicchetti-Allison weights^24^. Kappa was interpreted as 0, “no agreement”, 0.10–0.20, “slight”, 0.21–0.40, “fair”, 0.41–0.60, “moderate”, 0.61–0.80, “substantial”, and 0.81–0.99, “almost perfect agreement”^25^. A secondary outcome assessed the intraobserver concordance for observers 1 and 2, who re-read the subset of 34 participants. All analyses were performed using “vcd” and “grid” packages in R Version 1.4.1106 © 2009–2021 RStudio, PBC^26^.

## Results

Comparing the randomly selected subset of 425 participants with the whole cohort (4,338), we observed that the health characteristics of the subset were similar to those of the cohort (Table 1). Of note, we observed high rates of all SLD-related chronic diseases for both groups: overweight/obesity (61.5% in the cohort and 65.2% in the subset), severe obesity (28.5% and 25.6%, respectively), diabetes (25.7% and 25.1%, respectively), hypertension (81.3% and 79.7%, respectively), and an elevated percentage of high waist circumferences (94.9% and 95.7%, respectively, with ≥ 80 cm waist circumference), as expected since women with gallstones are a high-risk population. In particular, we found high rates of moderate/severe ultrasound-detected liver steatosis (Table 1).

**Table 1:** Health profile of the Chile Bils participants and the re-evaluated subset.

Health profile	Chile BiLS cohort (n = 4338)	Subset (n = 425)
Age, mean + SD	59.26 (6.57)	59.14 (6.44)
Education level, n (%)		
< 8 years	2259 (52)	218 (51.7)
9–12 years	1624 (37.4)	171 (40.5)
> 13 years	377 (8.8)	33 (7.8)
Missing values	78 (1.8)	3 (0.7)
Body mass index, n (%)^a^		
Normal	374 (8.6)	36 (9)
Overweight/obesity	2670 (61.5)	277 (65.2)
Severe obesity	1237 (28.5)	109 (25.6)
Missing values	57 (1.3)	3 (0.7)
Diabetes, n (%)^b^		
Yes	1113 (25.7)	107 (25.1)
No	3154 (72.7)	316 (74.4)
Missing values	71 (1.6)	2 (0.5)
High blood pressure, n (%)^c^		
Yes	3528 (81.3)	339 (79.7)
No	737 (17)	84 (19.8)
Missing values	73 (1.7)	2 (0.5)
Waist circumference, n (%)^d^		
≥ 80 cm	4115 (94.9)	407 (95.7)
< 80 cm	172 (3.9)	16 (3.8)
Missing values	51 (1.2)	2 (0.5)
Liver steatosis, n (%)^e^		
None	1096 (25.3)	98 (23.1)
Mild	1150 (26.5)	118 (27.8)
Moderate/severe	2090 (48.2)	209 (49.2)
Missing values	2 (0.1)	0 (0)

^a^Body mass index (BMI) is presented in kg/m^2^ 18–24 kg/m^2^: normal, 25–34 kg/m^2^: overweight/obesity, and 35– > 40 kg/m^2^: severe obesity.

^b^Diagnosed with diabetes by a physician or in diabetes treatment (self-report).

^c^Blood pressure ≥ 130/85 mmHg or in treatment for hypertension.

^d^Waist circumference ≥ 80 cm, based on the criteria for steatotic liver disease (SLD)^3^.

^e^Based on the original readings by radiology technicians.

**Fig. 3:**
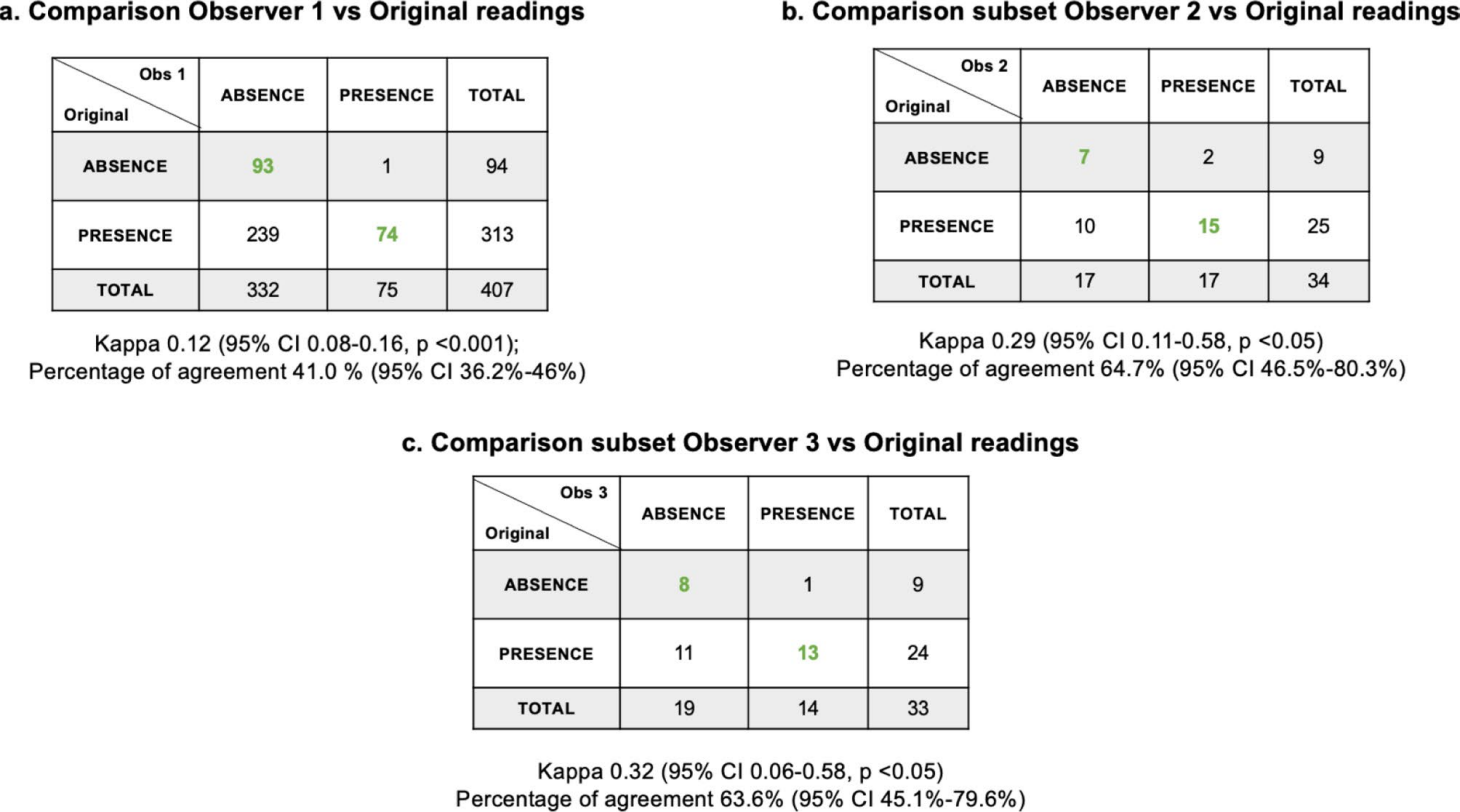
Inter-observer agreement for presence/absence of liver steatosis between the observers. (**a**) Agreement of observer 1 (radiologist) against 407 original readings (observer 1 excluded 18 images). (**b**) Agreement between observer 2 (first Chilean observer) and the subset of 34 images. (**c**) Comparison of the observer 3 (second Chilean technician) and the subset of 33 images (observer 3 excluded one image).

Of 425 selected participants, 18 (4.2%) were excluded by observer 1 because the kidney and liver were not on the same plane in the ultrasound image. Among the 407 participants compared in the classification of presence versus absence of steatosis, the agreement between observer 1 and the original reading was slight, with a kappa of 0.12 (95% CI 0.08–0.16, *p* < 0.001) and a percent agreement of 41.0% (95% CI 36.2–46%) (Fig. 3a). Significant discrepancies were identified in 239 (58.7%) individuals whom observer 1 classified as having “absence” of steatosis, while the original readings indicated “presence” of steatosis (Fig. 3a).

For the duplicate set of 34 participants, we compared the inter-observer agreement for the presence versus absence of steatosis between the two Chilean radiology technicians (observers 2 and 3) and the original readings. Observer 2 had a fair agreement (kappa: 0.29 (95% CI 0.11–0.58, *p* < 0.05), with 64.7% (95% CI 46.5–80.3%) agreement (Fig. 3b). Observer 3, who excluded 1 (2.9%) image because the kidney and liver were not on the same plane, had a fair agreement (kappa: 0.32, 95% CI 0.06–0.58, *p* < 0.05), with 63.6% (95% CI 45.1–79.6%) agreement (Fig. 3c). Both Chilean radiology technicians had discrepancies compared to the original readings, categorizing 10 (29.4%) and 11 (33.3%) individuals, respectively, as having no liver steatosis, while the original readings classified them as having liver steatosis (Figs. 3b–c).

**Fig. 4:**
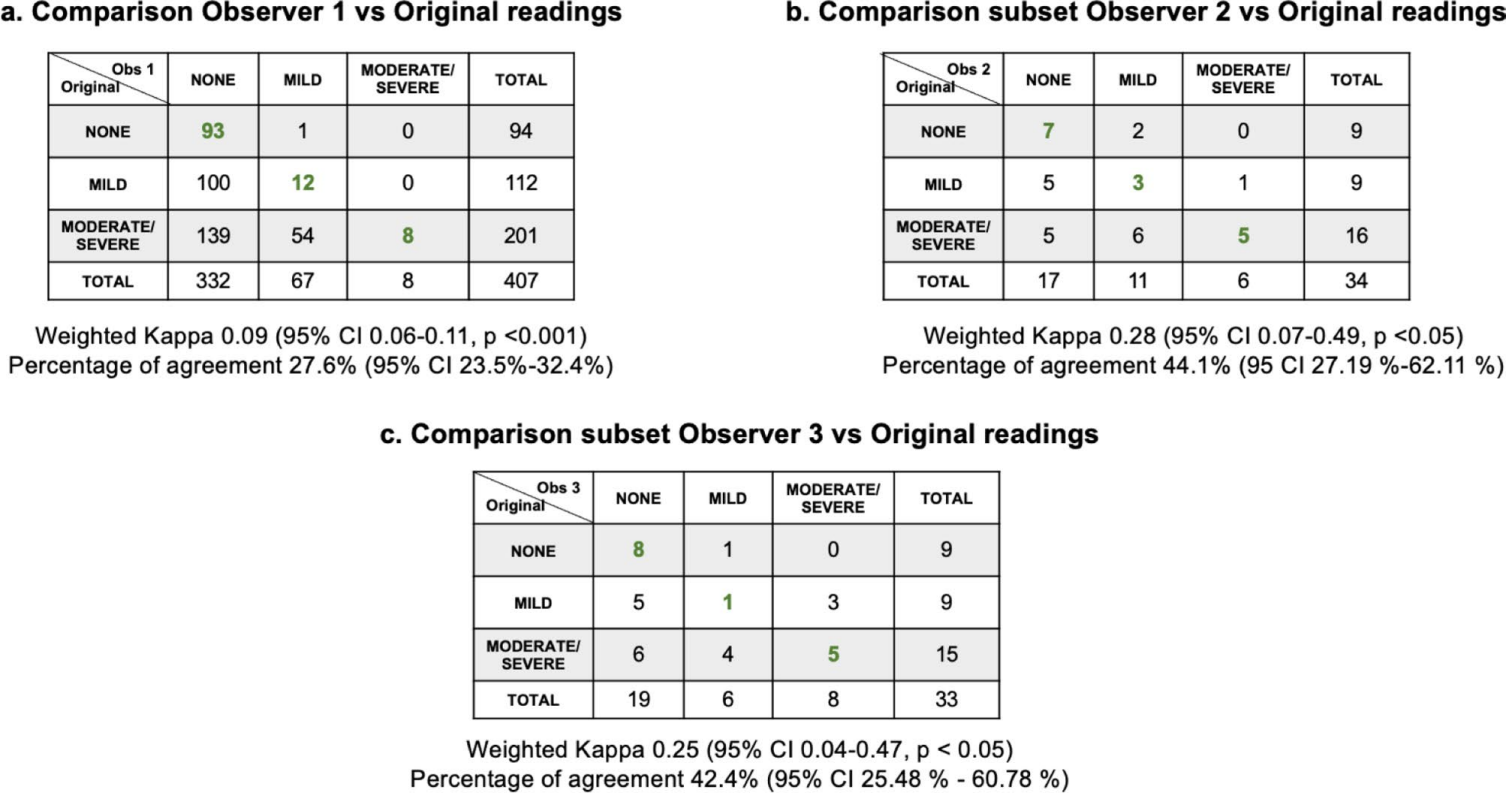
Inter-observer agreement of steatosis severity (none, mild, moderate/severe) between the observers. (**a**) The agreement of observer 1 (radiologist) against the 407 original readings (observer 1 excluded 18 images). (**b**) Agreement between observer 2 (first Chilean observer) and the 34 duplicated subsets. (**c**) Comparison of the observer 3 (second Chilean technician) and the original readings (observer 3 excluded one image).

The findings for steatosis level (none, mild, moderate/severe) were similar to those for presence/absence: observer 1 had a slight agreement (weighted kappa: 0.09, 95% CI 0.06–0.11, *p* < 0.001; percent agreement: 27.6%, 95% CI 23.5–32.4%, Fig. 4a) compared with the original readings. For observers 2 and 3, agreement was fair (weighted kappa: 0.28, 95% CI 0.07–0.49, *p* < 0.05; percent agreement: 44.1% (95 CI 27.19–62.11%); and weighted kappa: 0.25, 95% CI 0.04–0.47, *p* < 0.05; percent agreement: 42.4%, 95% CI 25.48–60.78%, respectively) (Fig. 4b–c). Observer 1 classified 100 (24.6%) participants as “none” who were initially classified as “mild” and 139 (32.9%) participants as “none” who were initially classified as “moderate/severe” (Fig. 4a). For observers 2 and 3, discrepancies were related to the classification of “none” versus “mild” [5 (14.7%) and 5 (15.2%), respectively] and “none” versus “moderate/severe” [5 (14.7%) and 6 (18.2%), respectively] (Fig. 4b–c).

**Fig. 5:**
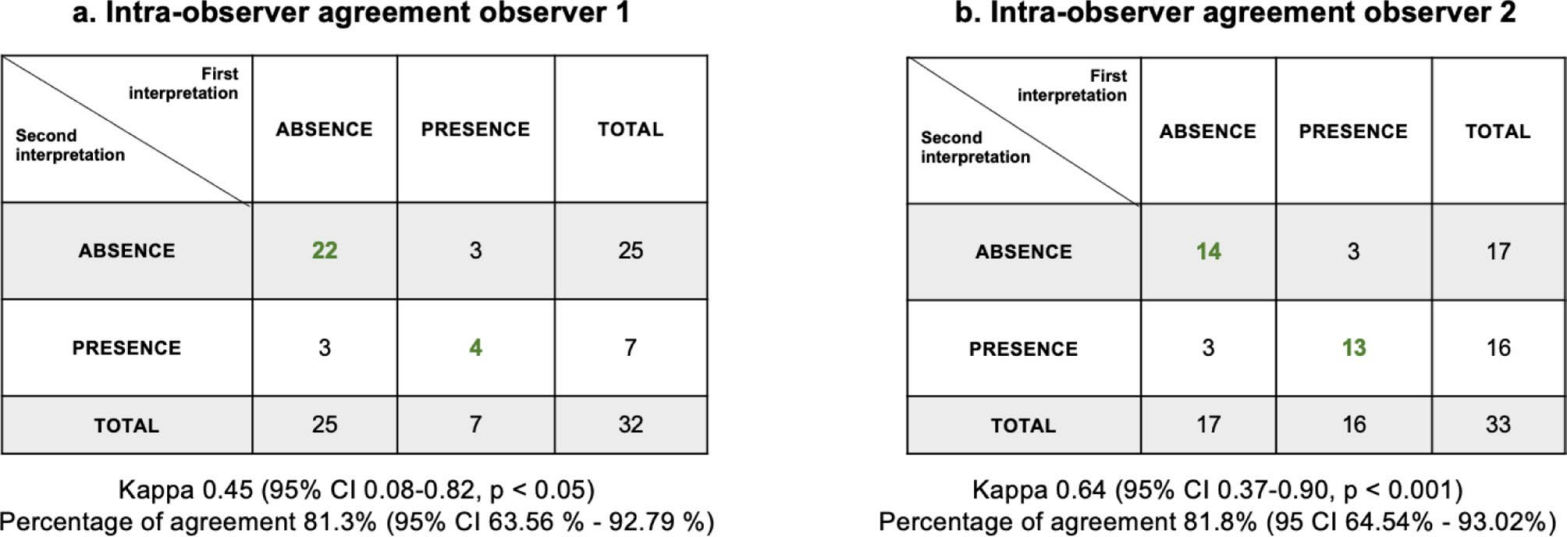
Intra-observer agreement for presence/absence of liver steatosis for radiologist (Observer 1) and Chilean technician (Observer 2). (**a**) Agreement between the first interpretation of the subset and the second interpretation of observer 1 (radiologist, excluded 2 images). (**b**) Comparison of the first and second interpretations of the subset ultrasound of the observer 2 (Chilean technician, excluded 1 image).

**Fig. 6:**
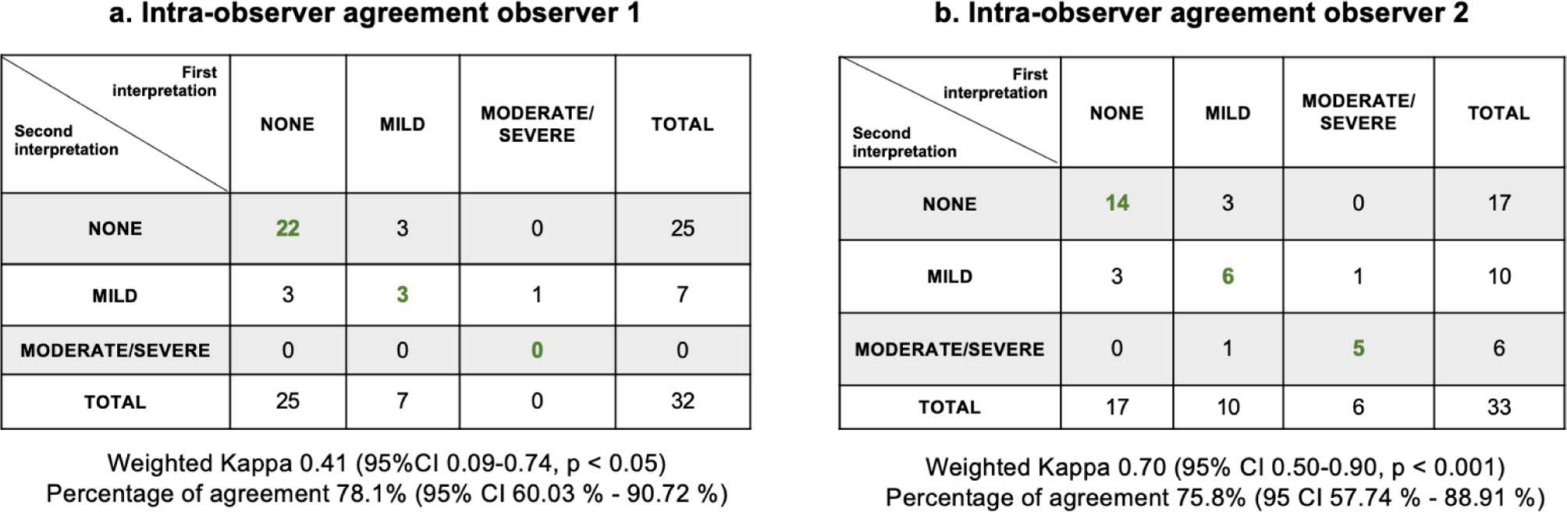
Intra-observer agreement of steatosis severity (none, mild, moderate/severe) of radiologist (observer 1) and Chilean technician (observer 2). (**a**) Agreement between the first interpretaion of the subset and the second interpretaion of observer 1 (radiologist, excluded 2 images). (**b**) Comparison of the first and second interpretaions of the subset ultrasound of the observer 2 (Chilean technician, excluded 1 image).

Regarding the intra-observer agreement, observer 1 excluded 2 (5.9%) images of the 34 participants included for re-review. For the 32 participants re-reviewed, agreement for presence vs. absence of liver steatosis was moderate, with a kappa of 0.45 (95% CI 0.08–0.82, *p* < 0.05) and a percent agreement of 81.3% (95% CI 63.56–92.79%) (Fig. 5a). Observer 2 excluded 1 (2.9%) image. Of the 33 participants re-reviewed, intra-observer agreement was substantial, with a kappa of 0.64 (95% CI 0.37–0.90, *p* < 0.001) and a percent agreement of 81.8% (95 CI 64.54–93.02%) (Fig. 5b). Discrepancies for both observers 1 and 2 were related to the categories of “none” versus “mild” (Fig. 6a–b).

## Discussion

The increase in SLD rates worldwide and their complications, such as fibrosis, cirrhosis, and cancer^27^, raises the importance of early detection and management. While elastography (e.g., FibroScan) is recommended, it is not available in many countries or regions due to its high costs^28^, highlighting that ultrasonography is one of the most accessible and recognized screening techniques for SLD diagnoses^12,29^, particularly in low and middle-income countries such as Chile.

Early detection of hepatic steatosis is clinically crucial for timely risk stratification, preventive measures, and appropriate clinical interventions.

However, ultrasound has limitations in its interpretation, mainly because it is highly operator-dependent; comparing two or more interpreters is essential to ensure a correct diagnosis. Our findings indicate poor inter-observer agreement, especially between the radiologist (observer 1) and the original readings, particularly in differentiating between “absence” versus “presence” of steatosis. This variability is clinically relevant as accurate early detection impacts timing and clinical interventions. In the early stages of SLD, especially in MASLD, where patients have mild steatosis, management is less invasive; lifestyle modifications, such as diet and exercise, can improve hepatic steatosis in most cases^15^. As the disease progresses, not only does steatosis progress, but also fibrosis appears, identifying it as metabolic dysfunction-associated steatohepatitis (MASH). MASH has less effective treatments. Studies have shown that improvement in MASH can be achieved if patients lose ≥ 10% of their body mass^15,30,31^, and due to the difficulties of losing that amount of weight, only 10–20% of the patients can achieve it^15,31^. On the other hand, more aggressive treatment, such as medication, has not yet proven to be highly effective in MASH^32^. This highlights the importance of a correct and early diagnosis; since no effective intervention has been demonstrated for patients with MASH, we need to identify the high-risk patients who can significantly benefit from an early, effective, non-invasive intervention.

Across the three observers, discrepancies were predominantly noted between the “none” and “mild” classifications. Such findings align with previous studies reporting lower ultrasound sensitivity in discriminating between none and mild steatosis^12^, with low sensitivity and specificity (60% and 84%, respectively). This reduced accuracy can be attributed to lower liver fat levels, making it difficult to determine if liver steatosis is present^29,33^. Recognizing this limitation is clinically significant as mild steatosis may often be underestimated, thereby delaying necessary preventive interventions.

Multiple factors likely contribute to discrepancies between observer 1 and the original findings. First, the cohort had a high prevalence of obesity (25.6% of the participants in the subset have a BMI of over 35 kg/m^2^, and 96% have a high waist circumference), which can interfere with the image quality. Studies have shown that obese patients have a more significant amount of abdominal adiposity, affecting ultrasound performance^12,34^. Heinitz et al. 2023 showed that ultrasound has a worse image quality in patients with a BMI above 35 kg/m^2^, which, in consequence, could affect image interpretation^35^. Secondly, observer 1 reviewed still images the radiology technicians took in the field instead of videos or conducting a real-time examination. Stored images limited the reviewer to examining one single plane, preventing further findings and exploration that would help the disease diagnosis. This limitation is highlighted by a study of interobserver agreement between three radiologists with over eight years of experience in ultrasonography. In this study, the radiologists reviewed still ultrasound images and did not produce high interobserver agreement, but only fair to moderate agreement^36^. Thus, the accuracy of the diagnosis could be affected not only by the degree of steatosis, which is a known limitation of ultrasound, but also by the material available for review, since real-time ultrasound gives a better perspective of the steatosis in the liver. A study comparing liver steatosis diagnosis in real-time ultrasound vs. liver biopsy showed that when liver steatosis exceeded 20%, the sensitivity and specificity of ultrasound in real-time increased to 100% and 90%, respectively^33^.

On the other hand, we hypothesize that the Chilean observers had fewer discrepancies with the original readings because all Chile BiLS radiology technicians, including those who conducted the original ultrasound examinations, received the same standardized training. Using the same ultrasound instruments, regularly performing the examinations in the same environments, following the same protocol, and capturing the images using established procedures could affect image interpretation. Although the intra-observer agreement for observers 1 and 2 was higher than the inter-observer agreement, observer 1’s (radiologist) readings showed moderate agreement between the first and second interpretations. In contrast, observer 2 (Chilean radiology technician) obtained substantial agreement. This result is not surprising since, as shown in a previous study, the chances of discrepancies in interpretation are higher between individuals than with oneself^36^.

Even though interobserver agreements for the three observers were not higher than moderate, the health profile of the Chile BiLS participants suggests a notably high prevalence of steatotic liver disease (SLD) in the cohort. Epidemiological studies have shown that subcategories of SLD, such as MASLD and MetALD, are the most prevalent and are strongly related to metabolic syndrome^3,27^. Main risk factors associated with MASLD and MetALD are obesity, diabetes, and hypertension^37,38^. Studies have shown that 65% of obese patients have MASLD, while 70% of patients with a diagnosis of type 2 diabetes have MASLD^39^. In Chile, previous reports from the 2017 Chilean National Health Survey showed that women over age 50 had high rates of overweight (43.6%) and obesity (41.7%), high hypertension (27.7%), and type 2 diabetes (14.0%)^40^. These data are similar to what we found in the Chile BiLS cohort. Given these substantial burdens of obesity, diabetes, and related metabolic conditions, the high prevalence of SLD identified by Chile BiLS radiology technicians seems reasonable. It underscores the importance of targeted screening and monitoring strategies in high-risk populations. Studies have shown the importance of SLD screening in populations with a high burden of obesity and diabetes, because the coexistence of this condition with SLD has a higher risk of disease progression^41^. Although the Chile BiLS cohort was initially designed to study gallbladder disease and cancer, its detailed characterization of metabolic risk factors and comprehensive ultrasound assessments provide an invaluable opportunity to explore SLD screening and its clinical implications within a high-risk group, thereby contributing important insights that may inform preventive strategies in similarly vulnerable populations worldwide.

Finally, we acknowledge several limitations of our study. First, the primary purpose of the Chile BiLS cohort was to study gallbladder disease and cancer, not SLD. Although SLD was a secondary outcome, the radiology technicians were trained to fully assess the hepatobiliary system. Still, it is possible that some ultrasound images may have focused more on gallbladder visualization than on optimal liver imaging. Second, our intra-observer (and for observers 2 and 3, and inter-observer) agreement analyses were based on a relatively small sample, leading to imprecision in the estimates. However, as shown in other studies and guidelines^42^, this sample size is sufficient to perform a robust statistical analysis and offers useful insight into the challenges of observer agreement^42^. Despite these limitations, our findings offer meaningful insight into the consistency and challenges of ultrasound-based diagnosis of SLD in a high-risk population and reinforce the importance of standardized training and quality control in implementing imaging-based screening strategies, particularly in resource-limited settings.

## Conclusion

In summary, our study emphasizes the importance of evaluating inter- and intra-observer agreement to optimize the reliability of ultrasound-based diagnosis of SLD, particularly given the critical role of ultrasound in early detection of SLD in resource-limited settings. The notably high prevalence of SLD identified by radiology technicians in the Chile BiLS cohort aligns closely with the participants’ high-risk metabolic profile, supporting the validity of ultrasound as an effective screening tool when standardized training is applied. Our findings reinforce the necessity of implementing targeted SLD screening programs with rigorous quality control and suggest that broader integration of trained ultrasound operators could substantially improve early detection efforts.

## Data Availability

Data relevant to the analysis but excluding sensitive information, such as Mapuche status, will be available for all participants who consented to data sharing. Contact Dr. Catterina Ferreccio (cferrecr@uc.cl) or Claudia Marco (cmarco@uc.cl) for data access.
